# Contribution of influenza to acute exacerbations of chronic obstructive pulmonary disease in Kashmir, India, 2010–2012

**DOI:** 10.1111/irv.12291

**Published:** 2014-10-21

**Authors:** Parvaiz A Koul, Umar H Khan, Romana Asad, Rubaya Yousuf, Shobha Broor, Renu B Lal, Fatimah S Dawood

**Affiliations:** a Department of Internal & Pulmonary Medicine, Sher-i-Kashmir Institute of Medical SciencesSrinagar, India; b All India Institute of Medical SciencesNew Delhi, India; c Influenza Division, Centers for Disease Control and Prevention (CDC)Atlanta, GA, USA

**Keywords:** Chronic obstructive lung disease, chronic obstructive, hospitalization, influenza viruses, pulmonary disease

## Abstract

We estimate the contribution of influenza to hospitalizations for acute exacerbations of chronic obstructive pulmonary disease (AECOPD) in Kashmir, India. Prospective surveillance for influenza among patients hospitalized with AECOPD was conducted at a tertiary care hospital. Patients had clinical data collected and nasal/throat swabs tested for influenza viruses. Outcomes among patients with and without influenza were compared with logistic regression adjusting for age and underlying conditions. During October 2010–September 2012, 498 patients hospitalized with AECOPD were enrolled, of whom 40 (8%) had received influenza vaccine. Forty (8%) had influenza; influenza virus detection peaked in winter (January–March). Patients with influenza were more likely to die during hospitalization (adjusted OR 3·4, CI 1·0–11·4) than those without.

## Introduction

Globally, an estimated 64 million people have chronic obstructive pulmonary disease (COPD), with 90% of COPD deaths occurring in low- and middle-income countries.^1^ In India, estimates of COPD prevalence in India have varied from 2 to 20% depending upon method of diagnosis. In Kashmir, India, the prevalence of spirometrically diagnosed COPD is 17% in males and 15% in females aged ≥40 years.^2^ Against a backdrop of high COPD prevalence, measures to prevent COPD exacerbations in India would have substantial public health impact. However, seasonal influenza vaccines are not routinely recommended for any target group in India due in part to a lack of national data on influenza.^3^ We used data from prospective surveillance for influenza among patients hospitalized with acute exacerbations of COPD (AECOPD) to estimate the contribution of influenza to the overall burden of AECOPD and describe the epidemiology of AECOPD at a tertiary care hospital in Kashmir, India.

## Methods

Sher-i-Kashmir Institute of Medical Sciences (SKIMS) is the main tertiary care hospital for persons with COPD in Kashmir, India and has 650 beds and an intensive care unit. During October 2010–September 2012, surveillance was conducted at SKIMS for patients aged ≥40 years hospitalized with AECOPD. AECOPD was defined as ≥2 major symptoms (increased dyspnea, sputum purulence, or sputum amount) or ≥1 major and ≥1 minor symptom (nasal discharge/congestion, wheezing, sore throat, or cough) for ≥2 consecutive days in a patient with COPD.^4^,^5^ Data were collected using a standardized case report form through interview of consenting patients or their family members and review of patients' medical records. Surveillance staff also telephoned patients or their families at least 30 days after hospital discharge to collect data on readmission or death within 30 days after discharge.

### Laboratory specimen collection and testing

Nasal and throat swabs were collected from participants at enrollment for testing by reverse-transcription polymerase chain reaction for influenza A and B viruses, and those positive for influenza A viruses were further subtyped for A/H1N1pdm09, A/H3N2, and seasonal H1N1 using US Centers for Disease Control and Prevention (CDC) protocols.

### Human subjects review

The surveillance protocol was reviewed and approved by the institutional review board of SKIMS. The protocol was also reviewed at the US CDC and determined to be for public health surveillance purposes exempted from further CDC IRB review.

### Data analysis

Frequencies were calculated to describe baseline characteristics, treatment, and outcomes among patients. Illness outcomes (i.e., readmission within 30 days of discharge and death during or within 30 days after hospitalization) were compared among those with and without influenza in multivariate analysis using logistic regression to adjust for age and presence of ≥1 underlying medical conditions. Statistical analyses were conducted using SAS version 9.1 (SAS Institute Inc., Cary, NC, USA).

## Results

During October 2010–September 2012, 498 patients hospitalized with AECOPD were enrolled. Of these, 308 (62%) patients were male and 400 (80%) had ≥1 additional underlying medical condition; hypertension (58%) and heart disease (19%) were the most common conditions (Table 1). Adverse environmental exposures were frequent with 66 (13%) patients reporting current tobacco use, 118 (24%) reporting secondhand tobacco smoke exposure, and 406 (82%) reporting use of indoor kitchens, 121 (30%) of whom used biomass fuels (i.e., wood, crop residues, or dung). Only 40 (8%) patients reported receiving seasonal trivalent influenza vaccine during the preceding year.

**Table 1:** Baseline characteristics of patients hospitalized with acute exacerbations of COPD, Kashmir, October 2010–September 2012 (*N* = 498)

	*n*	%
Male	308	62
Age group (years)		
≤39	1	0
40–59	97	19
60–69	183	37
≥70	217	44
Rural	299	60
Urban	199	40
≥1 additional underlying medical condition	400	80
Environmental exposures		
Current smoker	66	13
Secondhand smoke exposure	118	24
Indoor kitchen	406	82
Biomass fuels for cooking*	146	29
Biomass fuels for personal (*kangri*) or space heaters (*bukhari*)**	461	93
Chronic supplemental oxygen	167	34
Chronic steroid therapy	16	3
Received seasonal influenza vaccine during past year	40	8
Median symptom onset to specimen collection (days)	8	IQR 5–12
Influenza virus positive	40	8

* Biomass fuels for cooking include wood, cow dung, and crop residue.

** Biomass fuels for heating include wood or charcoal.

**Figure 1:**
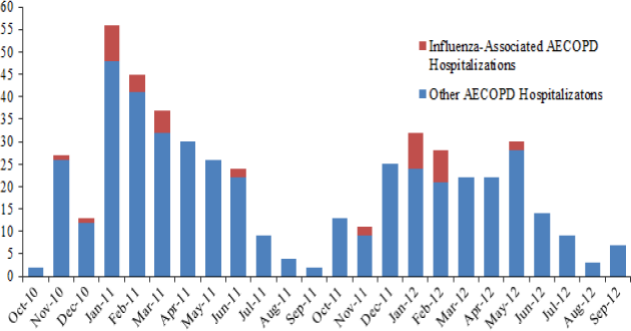
Acute exacerbation of chronic obstructive pulmonary disease hospitalizations by month, Kashmir, India, October 2010-September 2012.

The median time from symptom onset to respiratory specimen collection was 8 days (IQR 5–12 days). Overall, 40 (8%) patients had respiratory specimens positive for influenza viruses, of which 39 were subtyped (12 (31%) with A(H1N1)pdm09, 17 (44%) with A(H3N2), and 10 (26%) with B). Detection of influenza viruses and overall number of exacerbations peaked during January–March 2011 and January–February 2012; during peak periods, influenza was associated with 14 and 33% of AECOPD hospitalizations in 2011 and 2012, respectively (Figure 1). The proportion of patients hospitalized with influenza-associated AECOPD was similar during October 2010–September 2011 and October 2011–September 2012 (21/280 (8%) versus 19/218 (9%), *P* = 0·6).

Fifty-four (11%) patients with AECOPD required non-invasive mechanical ventilation with continuous positive airway pressure and 5 (1%) required invasive mechanical ventilation. While the majority of patients with AECOPD were treated with antibiotics (466/498, 94%) and steroids (352/498, 71%), only 1 (3%) of 40 patients with influenza-associated AECOPD was treated with an influenza antiviral medication.

Data on readmission within 30 days of hospital discharge and death during or within 30 days after hospital discharge were available for 317 patients (22 with influenza and 295 without influenza). The likelihood of readmission and death within 30 days of discharge was similar among patients with and without influenza after adjusting for age and underlying conditions [readmission 14% versus 4%, adjusted OR 3·3 (95% CI 0·9–12·8); death 17% versus 16%, adjusted OR 1·0 (0·3–3·8)]. However, patients with influenza were more likely to die during hospitalization [18% versus 6%, adjusted OR 3·4 (95% CI 1·0–11·4)].

## Discussion

During a 2-year period, influenza was associated with 8% of all hospitalizations for AECOPD at the main tertiary care hospital in Kashmir, India. The majority of influenza detection occurred during a defined 2- to 3-month period in the winter, when influenza was associated with 14–33% of hospitalizations for AECOPD. Few patients reported receiving influenza vaccines, and influenza antiviral treatment was uncommon. A substantial proportion of patients hospitalized with AECOPD required invasive or non-invasive mechanical ventilation and died during hospitalization or within 30 days after hospital discharge. Death during hospitalization was more common among patients with influenza than those without.

Our estimate of influenza virus detection prevalence among patients hospitalized with COPD exacerbations is consistent with previously published reports documenting influenza detection in 5–22% of patients hospitalized with exacerbations.^6^–^9^ Of the viral infections implicated in AECOPD, influenza is currently the only vaccine-preventable infection. The World Health Organization recommends influenza vaccination for groups at increased risk for severe influenza, such as persons with chronic lung disease, and influenza vaccination has been shown to reduce the incidence of COPD exacerbations.^10^,^11^ However, only 8% of patients in our study reported receiving influenza vaccine, consistent with limited data on uptake in other high-risk groups in India.^12^ Our findings, along with emerging data on influenza virus circulation and influenza-associated hospitalization incidence in other parts of India,^13^,^14^ suggest that influenza is a substantial contributor to severe respiratory illness in India and that recommendations for influenza vaccination in certain high-risk groups may be warranted.

Several issues should be considered when interpreting the results of our analysis. First, respiratory specimens were collected a median of 8 days from symptom onset, so we may have missed some cases of influenza and underestimated the prevalence of influenza among patients with COPD exacerbations. Second, we tested only for influenza viruses as influenza is currently the only vaccine-preventable respiratory virus infection.
